# Risk Factors Associated With Prolonged Antibiotic Use in Pediatric Bacterial Meningitis

**DOI:** 10.3389/fphar.2022.904322

**Published:** 2022-06-21

**Authors:** Cuiyao He, Xiaogang Hu, Tingsong Li, Qing Wu, Jisan Fan, Yan Zhou, Li Jiang, Siqi Hong, Yuanyuan Luo

**Affiliations:** ^1^ Department of Pharmacy, Ministry of Education Key Laboratory of Child Development and Disorders, Chongqing Key Laboratory of Pediatrics, National Clinical Research Center for Child Health and Disorders, Children’s Hospital of Chongqing Medical University, Chongqing, China; ^2^ Department of Pharmacy, Chongqing University Cancer Hospital, Chongqing, China; ^3^ Department of Rehabilitation, Ministry of Education Key Laboratory of Child Development and Disorders, Chongqing Key Laboratory of Pediatrics, National Clinical Research Center for Child Health and Disorders, Children’s Hospital of Chongqing Medical University, Chongqing, China; ^4^ Department of Neurology, Ministry of Education Key Laboratory of Child Development and Disorders, Chongqing Key Laboratory of Pediatrics, National Clinical Research Center for Child Health and Disorders, Children’s Hospital of Chongqing Medical University, Chongqing, China

**Keywords:** bacterial meningitis, children, augmented renal clearance, antibiotic, infectious disease

## Abstract

**Objectives:** To determine the risk factors associated with a prolonged antibiotic course for community-acquired bacterial meningitis (BM) in children.

**Methods:** This retrospective cohort study included children aged 1 month to 18 years with community-acquired BM due to a confirmed causative pathogen from 2011 to 2021. Patients were divided into an antibiotic prolongation group and a nonprolongation group according to whether the antibiotic course exceeded 2 weeks of the recommended course for the causative pathogen. Associations of important clinical characteristics and laboratory and other parameters with antibiotic prolongation were assessed using univariate and multivariable regression logistic analyses.

**Results:** In total, 107 patients were included in this study. Augmented renal clearance (ARC) (OR, 19.802; 95% CI, 7.178–54.628; *p* < 0.001) was associated with a prolonged antibiotic course; however, septic shock, causative pathogen, preadmission antibiotic use, peripheral white blood cell (WBC) count, initial cerebrospinal fluid (CSF) WBC count, CSF glucose, CSF protein, and surgical intervention were not associated with the prolonged antibiotic course. Patients with ARC had more total fever days (median time: 14 vs. 7.5 days), longer hospitalization (median time: 39 vs. 24 days), higher rates of complications (72.34% vs. 50.00%) and antibiotic adjustments (78.723% vs. 56.667%) than patients with normal renal function.

**Conclusion:** ARC is an independent risk factor for prolonged antibiotic use in children with community-acquired BM. ARC may be associated with longer fever and hospitalization durations, higher rates of complications and antibiotic adjustments.

## Introduction

Bacterial meningitis (BM) is a life-threatening infectious disease of the central nervous system in childhood. The mortality rate varies from 5% to 30%, and approximately 30–54% of survivors develop neurological sequelae (Namani et al., 2013). Antibiotics are the cornerstone of the pharmacological treatment of BM, and guidelines recommend standard antibiotic treatment protocols for different pathogens (van de Beek et al., 2012; van de Beek et al., 2016). We observed that the duration of antibiotic therapy in some children with BM caused by the positive pathogen often exceeded the recommended course, in spite of using the standard regimen. Prolonged antibiotic therapy leads to an increased length of hospital stay and antibiotic resistance, resulting in higher medical costs and a greater psychological burden. Therefore, the main purpose of our study was to identify factors associated with a prolonged antibiotic course for community-acquired BM in children.

The adequate concentration of antibiotics in the cerebrospinal fluid (CSF) is essential to BM treatment. The fact that augmented renal clearance (ARC) can decrease the concentration of the drug in the body is widely recognized, especially for drugs that are primarily eliminated by renal excretion (Udy et al., 2010; Van Der Heggen et al., 2019). Antibiotics such as β-lactams and vancomycin are the most important drugs in the treatment of community-acquired BM in pediatric patients, and they are mainly excreted by the kidneys (Kim, 2010; van de Beek et al., 2016). Accordingly, the presence of ARC in patients with BM can decrease some antibiotic concentrations in the body, inevitably affecting the treatment of the site of infection. Currently, there have been few studies about ARC effects on the antibiotic course and clinical outcomes for patients with BM, especially children (Lautrette et al., 2012; Xiao et al., 2022). This is the first study to consider ARC as a risk factor for prolonging antimicrobial agents used for BM, and it further elucidates whether ARC has an impact on other clinical indicators in pediatric community-acquired BM. This study will focus on whether the dosages of antimicrobial drugs administered according to current guidelines result in unsatisfactory treatment process of BM, that maybe be related to exposure to subtherapeutic concentrations because of ARC, while providing evidence of therapeutic optimization for these patients in the future.

## Methods

### Study Design and Population

Patients aged 1 month to 18 years with confirmed community-acquired BM admitted to the Department of Neurology, Children’s Hospital of Chongqing Medical University (CHCMU), from January 2011 to August 2021 were included in this study.

The inclusion criteria were as follows: 1) community-acquired BM with a confirmed causative pathogen (on the basis of positive identification of the causative organism from CSF or blood, via either bacterial culture or polymerase chain reaction (PCR); 2) aged between 1 month and 18 years; 3) liver and renal function testing performed within 72 h prior to the use of antimicrobial drugs; and 4) sensitive antimicrobial drugs based on pathogenic and drug sensitivity results.

The exclusion criteria were as follows: 1) hospital-acquired BM; 2) renal insufficiency or renal impairment that occurred during treatment; 3) hemodialysis; 4) various bacteria cultured from blood and CSF, with mixed bacterial infection unable to be ruled out clinically; 5) lack of use of sensitive antimicrobial drugs according to drug susceptibility testing; 6) an antibiotic therapeutic dose different from that recommended for pediatric community-acquired BM; 7) discharged from the hospital after giving up treatment, thus not allowing the clinical prognosis to be assessed; or 8) combined with primary immunodeficiency or anatomical abnormalities, such as CSF leak during the course of the disease.

Data from medical records was collected. The medical records of each patient included in the study were reviewed by two independent reviewers using a standardized data collection form. Discrepancies in the collected data were reconciled by a third reviewer. This study was approved by the Ethics Professional Committee of CHCMU (The Ethical Approval No:2021-422).

### Data Collection

To determine the risk factors associated with a prolonged antibiotic course for community-acquired BM in children, 23 potentially relevant variables were chosen for analysis. There were no missing data for the 23 variables extracted from each patient’s medical record, including age; sex; height; weight; premature status; maximum body temperature; neck stiffness; initial peripheral white blood cell (WBC) count and neutrophil percentage; CSF WBC count; CSF glucose concentration; CSF protein concentration; C-reactive protein (CRP), blood and CSF culture results (causative pathogens); estimated glomerular filtration rate (eGFR); creatinine, urea, albumin, alanine aminotransferase (ALT), and aspartate aminotransferase (AST) levels; preadmission antibiotic use; antibiotic adjustments during the course of the disease; and septic shock. To assess the efficacy of treatment, the efficacy evaluation indicators used in this study were duration of fever, length of hospitalization, presence of complications (subdural effusion, ventriculitis, hydrocephalus, cerebral edema, brain abscess, paralysis, facial palsy, actinic nerve palsy, or hearing loss), surgical intervention, antibiotic adjustment, and Glasgow Outcome Scale (GOS) at discharge.

The modified Schwartz formula (eGFR = k×height/Scr) was used to estimate the eGFR in patients younger than 17 years old, where k is 0.45 for infants aged <1 year, 0.55 for children aged <13 years and adolescent females, and 0.7 for adolescent males; and Scr is the serum creatinine concentration (mg/dl). Height is expressed in centimeters (cm).

### Study Subgroups

Patients were divided into an antibiotic nonprolongation group (ANPG) and an antibiotic prolongation group (APG). APG was the course of antibiotics 2 weeks longer than the recommended guideline, and ANPG was not exceed 2 weeks. The standard duration of antibiotic treatment for BM in this study was based on the European Society of Clinical Microbiology and Infectious Diseases guideline (van de Beek et al., 2016). The specific antibiotic regimens and the prolonged antibiotic course criteria for different pathogenic bacteria are detailed in Supplementary Table S1 (Chaudhuri et al., 2008; van de Beek et al., 2016). The main antibiotic doses used in this study were as follows: cefotaxime 75 mg/kg q6–8 h; ceftriaxone 50 mg/kg q12 h (maximum 2 g q12 h); ampicillin/amoxicillin 50 mg/kg q6 h, meropenem 40 mg/kg q8 h. Vancomycin 10–15 mg/kg q6 h to achieve serum trough concentrations of 15–20 μg/ml; If the serum concentration did not reach the target value, increased the dose of vancomycin to 18–20 mg/kg q6 h, or switched to linezolid 10 mg/kg q8 h (600 mg q12 h, >12 years), especially for *Staphylococcus aureus*, or rifampicin 10 mg/kg q12 h up to 600 mg/day, especially for *Streptococcus pneumoniae*. Chloramphenicol was considered when conventional antibiotic therapy was not effective.

### Definitions

1. Confirmed BM (Brouwer et al., 2010) was defined as the presence of clinical symptoms of meningitis and identification of bacteria directly (by culture from blood or CSF) in conjunction with CSF abnormalities suggestive of BM.
2. The GOS (Kastenbauer and Pfister, 2003) was used to assess the clinical status of patients on the day of discharge: 1, death; 2, persistent vegetative state; 3, severe disability; 4, moderate disability; 5, good recovery. A bad outcome was considered a GOS ≤1-4, and a good outcome was considered a GOS = 5.
3. In our study, elevated eGFR referred to eGFR_≥_160 ml/min/1.73 m^2^. Since ARC was defined as eGFR ≥160 ml/min/1.73 m^2^ in children (Hirai et al., 2016), elevated eGFR in our study was equivalent to ARC. eGFR was used as a continuous variable for the ROC curve and as a dichotomous variable in other analyses.
4. Complications were defined as subdural effusion, ventriculitis, hydrocephalus, cerebral edema, intracerebral hemorrhage, brain abscess, paralysis, facial palsy, actinic nerve palsy, or hearing loss that occurred during the acute course of BM.
5. Surgical interventions were defined as surgical procedures such as subdural puncture and surgical subdural drainage performed during the acute course of BM.
6. Antibiotic adjustment referred to the therapeutic strategy of increasing the drug dose, extending the infusion time and/or switching to other effective antimicrobial drugs if the clinical symptoms were not relieved or if the CSF parameters did not significantly improve during the treatment process with effective antimicrobial drugs, based on antibiotic susceptibility testing.
7. The antibiotic course in this study referred to the treatment course with sensitive antimicrobial drugs based on antibiotic susceptibility testing.
8. Prematurity was defined as birth at a gestational age<37 weeks (Blencowe et al., 2012).
9. Septic shock was defined as a hypotension (systolic blood pressure and/or mean blood pressure below the 5th percentile for age, secondary to sepsis (Parker et al., 2016).

### Data Analysis

Statistical analyses were conducted using SPSS version 26.0 (IBM, New York, NY, United States). Summary statistics for normally distributed quantitative variables are expressed as the means ± SDs, and nonnormally distributed variables are expressed as medians and interquartile ranges (IQRs). Categorical data are summarized by ratios and percentages. Differences in proportions were tested by the x^2^ test, Fisher’s test or Mann–Whitney *U* test (single ordinal contingency data). Logistic regression analysis was performed to analyze the association of the influencing factors and the outcome. The receiver operating curve (ROC)-derived optimal cutoff was determined at the maximal Youden Index. *p* values <0.05 were considered statistically significant.

## Results

### Analysis of the Demographic and Clinical Characteristics and Laboratory Parameters of the Two Groups

We identified 107 patients with community-acquired BM due to a confirmed causative pathogen at CHCMU during the study period, of whom 60 (56.07%) were males and 47 (43.93%) were females. The median age was 0.49 years, with an IQR of 0.20–2.41 years. Confirmed bacterial etiologies were as follows: 30 *Escherichia coli*; 37 *Streptococcus pneumoniae*; 8 *Listeria monocytogenes*; 12 GBS; 8 *Haemophilus influenzae* and 12 other bacteria (two *Streptococcus pyogenes*; two *Pseudomonas aeruginosa*; two *Neisseria meningitidis*; one *Capnocytophaga sputigena*; four *Staphylococcus*; and one *Streptococcus retardans*); more information on the characteristics are detailed in Table 1. Sixty-six patients (61.68%) were included in the antibiotic nonprolongation group, and 41 patients (38.32%) were included in the antibiotic prolonged group. Except for creatinine (*p* < 0.001), eGFR (*p* < 0.001), peripheral WBC (*p* < 0.028), and surgical intervention (*p* < 0.046), the differences in the other characteristics were not statistically significant.

**TABLE 1:** Analysis of the demographic and clinical characteristics and laboratory parameters of the two groups.

	ANPG (66, 61.68%)	APG (41, 38.32%)	χ^2^ value/Mann–Whitney U value	*p* value
Sex (Female, %)	30(45.455)	17(41.463)	0.164	0.686
Age (years)			1292.500	0.647
<1	41(62.121)	27(65.854)		
≥1, <7	20(30.303)	12(29.268)		
≥7, <13	5(7.576)	2(4.878)		
Preterm (yes, %)	63(95.455)	39(95.122)	——	1.000
eGFR (≥160, %)	13(19.697)	34(82.927)	41.049	<0.001
Height- for- age^a^ (<P3^b^, %)	7(10.606)	5(12.195)	——	1.000
Weight- for- age^c^ (<P3^b^, %)	6(9.091)	5(12.195)	——	0.745
Creatinine (umol/L)			19.777	<0.001
<14	5(7.576)	18(43.902)		
≥14, ≤60	61(92.424)	23(56.098)		
Urea (mmol/L)			0.909	0.340
<2.2	26(39.394)	20(48.780)		
≥2.2, ≤7.14	40(60.606)	21(51.220)		
ALT (>40 U/L, %)	22(33.333)	11(26.829)	0.502	0.479
AST (>45 U/L, %)	19(28.788)	11(26.829)	0.048	0.826
Albumin (g/L)			0.103	0.748
<38	47(71.212)	28(68.293)		
≥38, ≤55	19(28.788)	13(31.707)		
Septic shock (yes, %)	2(3.030)	5(12.195)	——	0.104
Maximum temperature(°C)			1188.000	0.184
<37.5	5(7.576)	1(2.439)		
≥37.5 <38	2(3.030)	1(2.439)		
≥38, <39	15(22.727)	7(17.073)		
≥39	44(66.667)	32(78.049)		
Neck stiffness (yes, %)	33(50.000)	20(48.780)	0.015	0.902
CFS glucose (mmol/L)			1157.000	0.179
≤1.11	22(33.333)	15(36.585)		
>1.11, ≤2	12(18.182)	14(34.146)		
>2	32(48.485)	12(29.268)		
CFS protein (g/L)			1104.500	0.082
≤1	21(31.818)	7(17.073)		
>1, <3	32(48.485)	22(53.659)		
≥3	13(19.697)	12(29.268)		
CFS WBC (per mm^3^)			1328.000	0.862
<100	12(18.182)	7(17.073)		
≥100, <1000	26(39.394)	16(39.024)		
≥1000, <10,000	28(42.424)	18(43.902)		
Blood WBC (10^9^/L)			1044.500	0.028
<4.4	8(12.121)	10(24.39)		
≥4.4, ≤11.9	17(25.758)	14(34.146)		
>11.9	41(62.121)	17(41.463)		
Percent neutrophils (%)			1276.500	0.518
<22	48(72.727)	32(78.049)		
≥22, ≤65	13(19.697)	7(17.073)		
>65	5(7.576)	2(4.878)		
CRP (≥8 mg/L, %)	61(92.424)	39(95.122)	——	0.705
Organism			——	0.411
* E. coli*	19(33.333)	11(36.667)		
* S. pneumoniae*	24(42.105)	13(43.333)		
* L. monocytogenes*	7(12.281)	1(3.333)		
GBS	7(12.281)	5(16.667)		
* H. influenzae*	3(5.263)	5(16.667)		
Other	6(10.526)	6(20.000)		
Preadmission antibiotics (yes, %)	56(84.848)	31(75.610)	1.420	0.233
Surgical intervention (yes, %)	14(21.212)	16(39.024)	3.977	0.046

Abbreviations: ANPG = antibiotic nonprolongation group, APG = antibiotic prolongation group, ALT = Alanine aminotransferase, AST = Aspartate aminotransferase, eGFR = estimated glomerular filtration rate, *E. coli* = *Escherichia coli*, S. *pneumoniae* = *Streptococcus pneumoniae*, *L. monocytogenes* = *Listeria* monocytogenes, GBS = Group *B Streptococcus, H.* influenzae = *Haemophilus* influenzae, CSF = Cerebrospinal fluid, WBC = White blood cell, CRP = C-reactive protein.

a Height-for-age referred to the level of height of children of the same age and gender.

b The 3rd percentile of height or weight of children of the same age and gender according to WHO standards.

c Weight of age referred to the level of weight of children of the same age and gender.

### Risk Factors Associated With a Prolonged Antibiotic Course

The univariate analysis without any correction showed that the rates of elevated eGFR (OR, 19.802; 95% CI, 7.178–54.628; *p* < 0.001), creatinine <14 μmol/L (OR, 9.548; 95% CI, 3.176–28.705; *p* < 0.001), CFS glucose (>1.11 mmol/L, ≤2 mmol/L) (OR, 3.111; 95% CI, 1.125–8.604; *p* = 0.029) and surgical intervention (OR, 2.377; 95% CI, 1.005–5.625; *p* = 0.049) were significantly different between the two groups.

To explore potential independent factors, further analysis was performed using multivariable analysis. In an analytical model incorporating all factors simultaneously, it was suggested that only elevated eGFR (OR, 258.531; 95% CI, 15.492–4314.476; *p* < 0.001) was a potential risk factor for prolonged antibiotic use. On this basis, stepwise regression analysis was also performed, and the results showed that only one influencing factor, elevated eGFR, was significant (OR, 19.802; 95% CI, 7.178–54.628; *p* < 0.001). All results in this section are detailed in Table 2.

**TABLE 2:** Associations between various clinical factors and prolonged antibiotic use in children with bacterial meningitis.

	Univariate analysis			Multivariable analysis			Stepwise regression analysis		
Risk factor	*p* value	OR	95% CI	*p* value	OR	95% CI	*p* value	OR	95% CI
Sex (Female)	0.686	0.850	0.387–1.869	0.836	1.250	0.152–10.261			
Age (years)									
≥7, <13 (ref)		1			1				
<1	0.568	1.646	0.298–9.105	0.403	5.500	0.101–298.682			
≥1, <7	0.657	1.500	0.251–8.977	0.801	0.617	0.014–26.542			
Premature	0.937	1.077	0.172–6.735	0.977	0.931	0.007–118.195			
Elevated eGFR	<0.001	19.802	7.178–54.628	<0.001	258.531	15.492–4314.476	<0.001	19.802	7.178–54.628
Height- for -age <P3^a^	0.800	1.171	0.346–3.966	0.575	2.482	0.104–59.399			
Weight -for -age <P3^a^	0.608	1.389	0.395–4.880	0.436	0.374	0.032–4.436			
Creatinine <14 umol/L	<0.001	9.548	3.176–28.705	0.551	0.395	0.019–8.367			
Urea <2.2 mmol/L	0.341	1.465	0.667–3.218	0.527	0.536	0.078–3.689			
ALT>40 U/L	0.480	0.733	0.310–1.733	0.432	0.320	0.019–5.487			
AST>45 U/L	0.826	0.907	0.379–2.170	0.543	0.480	0.045–5.086			
Albumin<38 g/L	0.749	0.871	0.373–2.030	0.460	0.486	0.072–3.289			
Septic shock	0.084	4.444	0.820–24.084	0.052	23.385	0.979–558.592			
Body temperature (°C)									
<37.5 (ref)		1			1				
≥37.5, <38	0.577	2.500	0.100–62.605	0.443	0.019	0–488.233			
≥38, <39	0.475	2.333	0.228–23.908	0.452	5.002	0.075–332.662			
≥39	0.249	3.636	0.405–32.648	0.723	2.346	0.021–263.918			
Neck stiffness	0.902	0.952	0.437–2.077	0.579	1.734	0.248–12.113			
CFS glucose (mmol/L)									
>2 (ref)		1			1				
>1.11, ≤2	0.029	3.111	1.125–8.604	0.117	5.763	0.646–51.412			
≤1.11	0.209	1.818	0.715–4.623	0.305	3.818	0.295–49.348			
CFS Protein (g/L)									
≤1 (ref)		1			1				
>1, <3	0.161	2.062	0.749–5.680	0.354	3.436	0.252–46.825			
≥3	0.085	2.769	0.867–8.840	0.127	9.676	0.526–178.112			
CFS WBC (/mm^3^)									
<100 (ref)		1			1				
≥100, <1000	0.925	1.055	0.344–3.237	0.506	0.316	0.011–9.423			
≥1000, <10,000	0.863	1.102	0.365–3.325	0.190	0.075	0.002–3.595			
Blood WBC (10^9^/L)									
4.4–11.9 (ref)		1			1				
<4.4	0.484	1.518	0.472–4.882	0.220	0.183	0.012–2.768			
>11.9	0.137	0.503	0.204–1.245	0.255	0.330	0.049–2.231			
Percent neutrophils (%)									
≥22, ≤65 (ref)		1			1				
<22	0.682	1.238	0.446–3.440	0.143	43.232	0.279–6698.441			
>65	0.757	0.743	0.113–4.867	0.537	3.593	0.062–208.637			
CRP ≥8 mg/L	0.586	1.598	0.295–8.648	0.799	0.563	0.007–46.545			
Organism									
Other (ref)		1			1				
*E. coli*	0.429	0.579	0.150–2.241	0.298	13.664	0.099–1880			
*S. pneumoniae*	0.362	0.542	0.145–2.023	0.549	0.381	0.016–8.942			
*L. monocytogenes*	0.109	0.143	0.013–1.546	0.173	0.051	0.001–3.709			
GBS	0.682	0.714	0.143–3.579	0.371	0.164	0.003–8.605			
*H. influenzae*	0.583	1.667	0.269–10.334	0.199	10.969	0.284–424.164			
Preadmission antibiotic	0.237	0.554	0.208–1.475	0.333	2.980	0.327–27.168			
Surgical intervention	0.049	2.377	1.005–5.625	0.375	2.909	0.275–30.763			

Abbreviations: ALT = Alanine aminotransferase, AST = Aspartate aminotransferase, eGFR = estimated glomerular filtration rate, *E. coli* = *Escherichia coli*, S. *pneumoniae* = *Streptococcus pneumoniae*, *L. monocytogenes* = *Listeria* monocytogenes, GBS = Group B *Streptococcus*, H. influenzae = *Haemophilus* influenzae, CSF = Cerebrospinal fluid, WBC = White blood cell, CRP = C-reactive protein, ref = reference.

a The 3rd percentile of height or weight of children of the same age and gender according to WHO standards.

### ROC Curve

**FIGURE 1:**
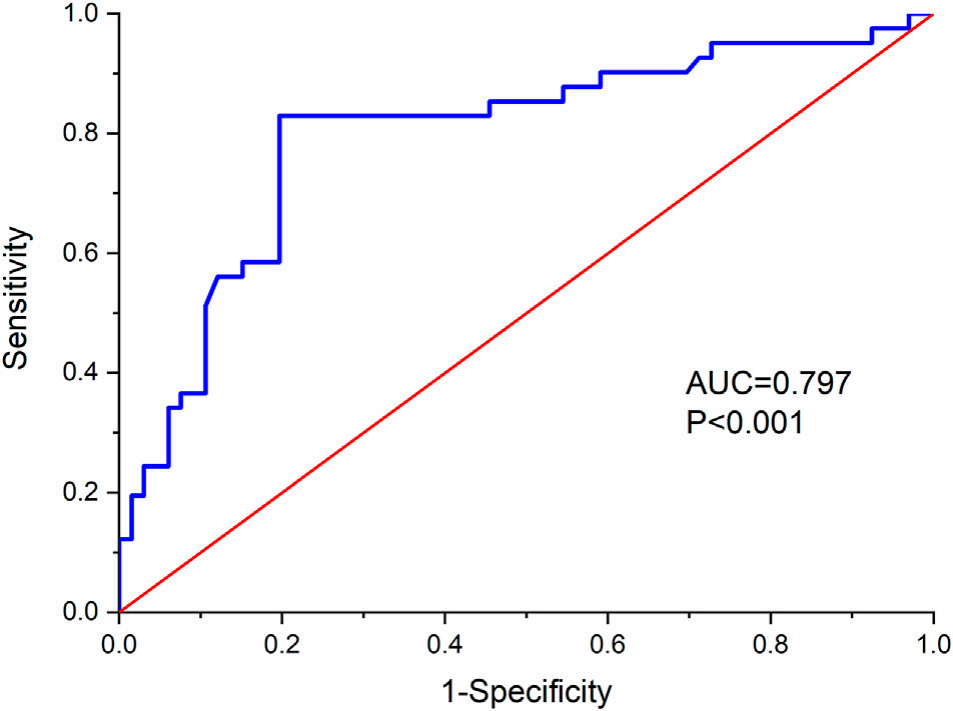
Receiver operating characteristic (ROC) curve.

The ROC curve was plotted with the actual measurement of eGFR (Figure 1), and the area under the curve (AUC) was 0.797. Further analysis showed that the maximum Youden index (0.632) was obtained when the eGFR = 159.84 (approximately equal to 160), suggesting that it is scientifically reasonable to assign 160 as the eGFR cutoff.

### Diagnostic Results

An eGFR = 160 ml/min/1.73 m^2^ and the diagnostic parameters obtained are shown in Table 3. An eGFR threshold of ≥160 ml/min/1.73 m^2^ had a sensitivity and specificity for antibiotic prolongation of 0.803 and 0.829, respectively. The positive predictive value and negative predictive value were 0.883 and 0.723, respectively, while the positive likelihood ratio and negative likelihood ratio were 4.703 and 0.238, respectively.

**TABLE 3:** The ability of eGFR (cut off 160 ml/min/1.73 m^2^) to classify and distinguish antibiotic prolongation.

Sensitivity	Specificity	Positive predictive value	Negative predictive value	Positive likelihood ratio	Negative likelihood ratio	Accuracy
0.803	0.829	0.883	0.723	4.703	0.238	0.813

### Clinical Efficacy Indicators in Different eGFR Subgroups

**FIGURE 2:**
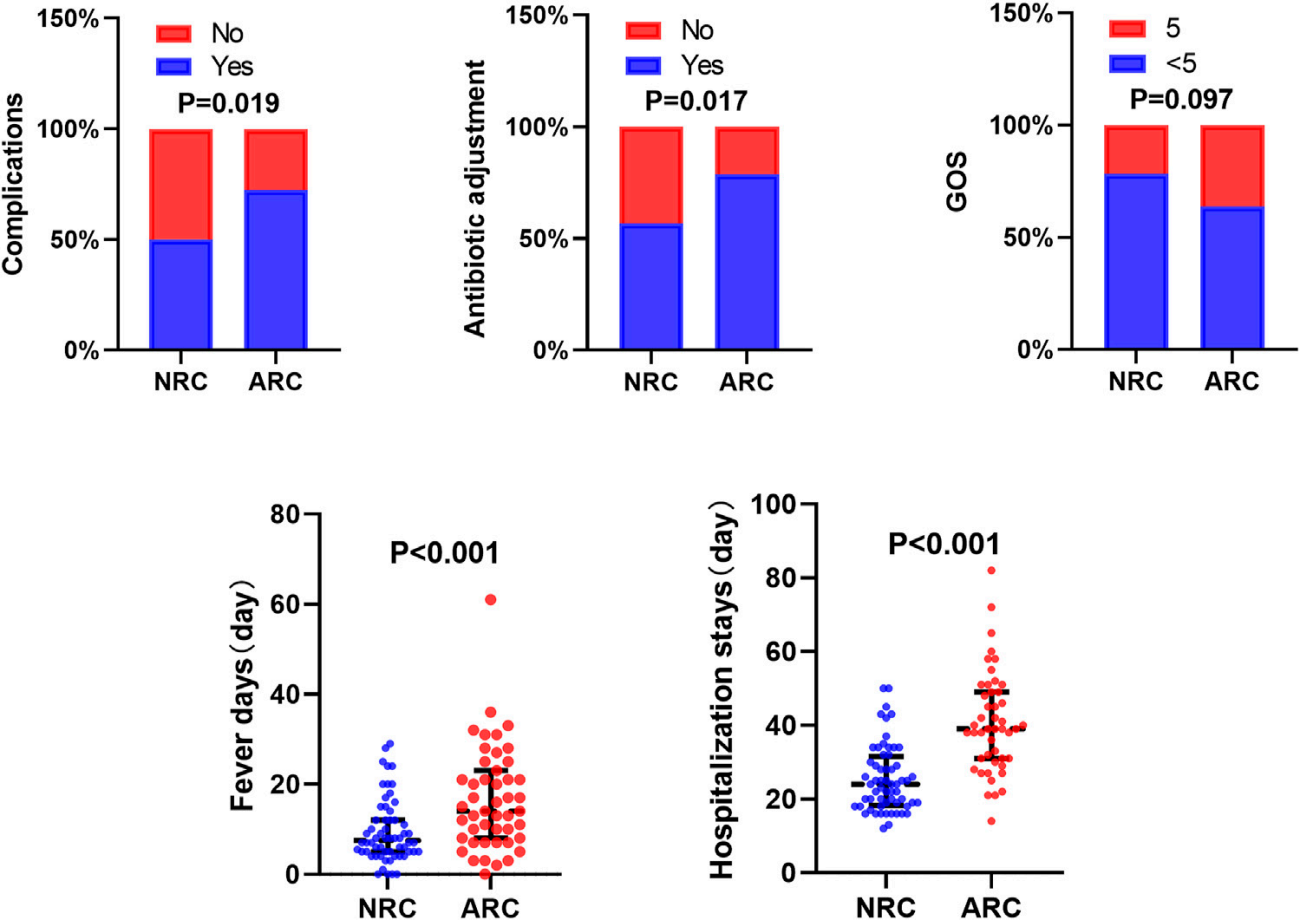
Clinical efficacy indicators in different eGFR subgroups.

The clinical efficacy indicators in the different eGFR subgroups were analyzed and are detailed in Figure 2. Patients were divided into an ARC group (eGFR_≥_160 ml/min/1.73 m^2^) and a normal renal clearance (NRC) group (eGFR<160 ml/min/1.73 m^2^). The analysis suggested that the group with an elevated eGFR had more total fever days (median time: 14 vs. 7.5 days), a longer total hospitalization stay (median time: 39 vs. 24 days) and higher rates of complications (72.34% vs. 50.00) and antibiotic adjustments (78.723% vs. 56.667%) than the NRC group. However, ARC cannot yet be considered to affect the prognosis of patients (*p* = 0.097).

## Discussion

We studied for the first time the risk factors for prolonged antibiotic treatment in pediatric patients with BM and found that ARC was an independent risk factor. Prolonged antibiotic therapy not only indicates longer hospital stays and higher treatment expenses but also serves as an indicator of an unsatisfactory treatment process for patients with BM (Peng et al., 2018). Current clinical research on BM has been focused on risk factors for unfavorable outcomes, such as neurological sequelae and death, but no studies on the factors influencing the course of antibiotic treatment have been conducted. We believed that the duration of antibiotic therapy was a very important indicator for evaluating the therapeutic efficacy against BM and therefore investigated risk factors for a prolonged course of antibiotic treatment in our study. In addition, with the development of precision medicine, treatment regimens for infectious diseases increasingly emphasize individualized drug delivery. It is known that the same standard anti-infective regimen used for different patients with the same disease may result in different therapeutic outcomes, which is closely related to different pathophysiological characteristics. Our study analyzed the risk factors for prolonged antibiotic therapy for pediatric BM, identified the specific pathophysiological characteristics of these patients, and further suggested that patients with these risk factors needed the antibiotic regimen to be optimized.

There were few studies on the correlation between ARC and clinical efficacy. Claus et al. (2013) found a higher antimicrobial therapeutic failure rate in the ARC group (27.3% vs. 12.9% *p* = 0.04) than in the non-ARC group. Carrié et al. (2018) found that ARC was associated with higher rates of underdosing, even when patients received high doses of continuously administered β-lactams. De Cock et al. (2015), through a population pharmacokinetics-pharmacodynamics modeling approach, reported clinical failure in the treatment of Enterobacteriaceae infections in critically ill children due to subtherapeutic concentrations of amoxicillin-clavulanic acid in the presence of ARC. These studies have preliminarily found an association of ARC with the failure of anti-infective therapy. Different from these studies, our study was conducted through rigorous selection of enrolled cases, especially the patients in our study were administered guideline-recommended treatment regimens. Unreasonable types, doses, and frequencies of antibiotics will inevitably affect the course and effectiveness of anti-infective treatment. Since this study aimed to clarify why antibiotic duration for BM caused by the same pathogen varied widely even under standard anti-infective regimens recommended by guidelines, we excluded the cases with irrational use of antibiotics through strict inclusion and exclusion criteria. And there was no difference in the percentage of pathogenic bacteria between the two groups of patients in baseline information, thus indicating the overall treatment regimen was essentially the same for both groups. Therefore, our study did not take antibiotic regimens as a risk factor for prolonged antibiotics analysis. And we used three different statistical methods, namely, univariate analysis, multivariate analysis and stepwise regression analysis, the final statistical analyses showed that only ARC was independently associated with antibiotic duration. Consequently, ARC was identified as an independent risk factor for prolonged antibiotic treatment of pediatric community-acquired BM.

The ARC phenomenon, in which renal function is significantly enhanced, usually occurs in critically ill patients (Bilbao-Meseguer et al., 2018; Van Der Heggen et al., 2019). Some studies have demonstrated several risk factors for ARC, including a younger age, severe neurologic injury, sepsis, trauma, and burns (Bilbao-Meseguer et al., 2018; Cook and Hatton-Kolpek, 2019). Lautrette et al. (2012) observed that ARC had an incidence of 25.0–47.0% in intensive care unit patients admitted with community-acquired BM. BM in children, a serious infectious disease of the central nervous system, is often comorbid with sepsis, and thus, these children are at high risk for the development of ARC and need more attention. ARC in adults is typically defined as creatinine clearance (CrCl) ≥ 130 ml/min/1.73 m^2^. However, the definition of ARC in children remains controversial (Bilbao-Meseguer et al., 2018). Hirai et al. (2016) defined ARC in children as an eGFR ≥160 ml/min/1.73 m^2^, which has been widely accepted. After our study concluded that ARC was an independent risk factor for prolonged antibiotic treatment, we further performed an analysis using a ROC diagnostic curve and demonstrated that the eGFR = 159.84 (approximately equal to 160) served as the ARC diagnostic value, corresponding to a sensitivity of 0.803 and a specificity of 0.829. These results confirm the reliability of an eGFR ≥160 ml/min/1.73 m^2^ as the criterion for determining ARC in children and provide a reference for ARC criteria in children, which is helpful for us to effectively identify ARC in patients in the clinic.

Many studies have confirmed that ARC is clearly associated with subtherapeutic antimicrobial concentrations of renally eliminated drugs (Hirai et al., 2016; Carrié et al., 2018; Cook and Hatton-Kolpek, 2019). Because of the specificity of the site of BM infection, the dosages of antimicrobial drugs used for infectious meningitis are widely higher than those usually prescribed, and a prompt use of effective antibiotic concentrations in CSF is of major concern for therapeutic success. If the antibiotic concentration in the CSF exceeds the minimum inhibitory concentration but does not reach the minimal bactericidal concentration, the bacteria are not quickly eliminated, which leads to a prolonged infection. We speculated that ARC could lead to accelerated excretion of antimicrobial drugs in patients with BM, with the risk of an insufficient dosage in CSF, which may lead to treatment failure. Fransson et al. (2021) reported a patient with intracranial infection who had subtherapeutic vancomycin and meropenem concentrations due to ARC and needed high doses of antibiotics to achieve therapeutic concentrations, this case report confirmed our guess. Ouchenir et al. (2017) found prolonged antibiotic use in several patients who developed complications due to GBS and *Escherichia coli* meningitis but did not elaborate on the correlation between long-term antimicrobial use and complications. Based on the above findings, we speculated that ARC not only led to a prolonged course of antibiotics but was also correlated with other clinical outcomes of BM. We then divided our study into ARC and non-ARC groups and found a significantly longer duration of fever, a longer length of the hospital stays, a larger number of antimicrobial adjustments, and a higher rate of complications in the ARC group than in the non-ARC group. Poor treatment outcomes were reflected not only by poor temperature control but also by higher complication rates due to uncontrolled infections.

As the existing recommended antimicrobial regimens could not achieve the desired pharmacokinetics-pharmacodynamics targets in critically ill patients with ARC, incremental dosing adjustment of antibiotics was frequently needed (Wong et al., 2018; Silva et al., 2022; Xiao et al., 2022). Special attention should also be paid to the use of antibiotics in children with severe infectious diseases in combination with ARC. Vancomycin is commonly used for pediatric BM, and our previous study has found that the vancomycin dose should be increased in pediatric patients with ARC, providing a dose reference for optimizing vancomycin dosing strategies (He et al., 2021). Grégoire et al. (2019) in a population PK study in adult patients with BM, highlighted the need for dose adjustment according to the eGFR and weight when using ceftriaxone to avoid underdosing using current guidelines, but the study was not conducted on pediatric patients with ARC. Our current study found that ARC significantly affected the course of antimicrobial therapy for pediatric BM and was associated with a higher incidence of complications. The reason for the unsatisfactory treatment in these patients with ARC is that it is difficult to achieve effective therapeutic concentrations of antibiotics locally in the infected area under the treatment regimens recommended by current guidelines with these specific pathophysiological characteristics. Thus, there is still a need for more research on optimal antibiotic regimens for children with BM combined with ARC to further improve clinical outcomes.

Because the study was a retrospective chart review, there were a few limitations. The definition of the prolonged antibiotic course criteria is not universally acknowledged. BM caused by multiple pathogens was included in this study, and the treatment regimen was different for each pathogen. Due to the limited samples for each pathogen, it was impossible to statistically analyze the effect of different treatment regimens for the same pathogen on antibiotic regimens. In future studies, we will increase the sample size for each pathogen and analyze the antibiotic regimen for each pathogen to further clarify whether it may lead to prolonged antibiotic therapy. The concentrations of antibiotic *in vivo* were the most direct indicators of the effect of ARC on drug metabolism in patients, however, it was not available in the study.

## Conclusion

This study first identified risk factors for prolonged antibiotic treatment of community-acquired BM in children. We found that ARC was an independent risk factor for prolonged antibiotic treatment of BM in children and determined that an eGFR ≥160 ml/min/1.73 m^2^ was an appropriate cutoff for the definition of ARC. ARC may be associated with a prolonged duration of fever, a prolonged length of stay, and increased rates of complications and antibiotic adjustments. This further clarifies the impact of ARC on the clinical efficacy of antibiotics against BM in children. Clinicians should optimize antibiotic regimens early in the treatment of childhood BM in patients with ARC to achieve a good outcome.

## Data Availability

The original contributions presented in the study are included in the article/Supplementary Material, further inquiries can be directed to the corresponding author.
