# Supplementary material for: Risk Factors Associated With Prolonged Antibiotic Use in Pediatric Bacterial Meningitis
Source: Front Pharmacol. 2022 Jun 21;13:904322. doi: 10.3389/fphar.2022.904322 (PMC9253569; doi:10.3389/fphar.2022.904322)
Supplement: Supplementary file 1 [file Table1.DOCX]

Supplementary Material

Table S 1: The specific antibiotic regimens and the prolonged antibiotic course criteria for different causative pathogens in our study

| **Pathogens** | **Antibiotic regimens** | **Recommended duration** | **Prolonged antibiotic course** |
| --- | --- | --- | --- |
| ***E. coli^a^*** |  |  |  |
| Third-generation cephalosporin susceptible | Ceftriaxone, cefotaxime, meropenem | At least 21 days | >35 days |
| Third-generation cephalosporin resistant | Meropenem | At least 21 days | >35 days |
| ***S. pneumoniae*** |  |  |  |
| Penicillin susceptible (MIC <0.1 μg/mL) | Penicillin, amoxicillin, ampicillin, ceftriaxone, cefotaxime | 10-14 days | >28 days |
| Penicillin resistant (MIC >0.1 μg/mL), third-generation cephalosporin susceptible (MIC <2 μg/mL) | Ceftriaxone, cefotaxime, cefepime, meropenem | 10-14 days | >28 days |
| Cephalosporin resistant (MIC≥ 2 μg/mL) | Vancomycin plus rifampicin, or vancomycin plus ceftriaxone or cefotaxime, or rifampicin plus ceftriaxone or cefotaxime; linezolid | 10-14 days | >28 days |
| ***L. monocytogenes*** | Amoxicillin, ampicillin, meropenem, linezolid | At least 21 days | >35 days |
| ***GBS^b^*** | Penicillin G, ampicillin, ceftriaxone, cefotaxime | At least 21 days | >35 days |
| ***H. influenzae*** |  |  |  |
| β-Lactamase negative | Amoxicillin, ampicillin, ceftriaxone, cefotaxime | 7-10 days | >24 days |
| β-Lactamase positive | Ceftriaxone, cefotaxime, cefepime, ciprofloxacin | 7-10 days | >24 days |
| β-Lactamase negative ampicillin resistant | Ceftriaxone or cefotaxime plus meropenem | 7-10 days | >24 days |
| ***Staphylococcus aureus*** |  |  |  |
| Methicillin sensitive | Flucloxacillin, vancomycin, linezolid, rifampicin^c^ | At least 14 days | >28 days |
| Methicillin resistant | Vancomycin, linezolid, rifampicin^c^ | At least 14 days | >28 days |
| Vancomycin resistant (MIC>2.0ug/ml) | Linezolid, rifampicin^c^ | At least 14 days | >28 days |

^a^ All *E. coli* in this study were sensitive to carbapenems

^b^ All *GBS* in this study were sensitive to penicillin

^c^Must not be used in monotherapy
